# Advanced Medical Image Analysis

**DOI:** 10.1155/2014/975372

**Published:** 2014-07-01

**Authors:** Rong Chen, Zhongqiu Wang, Yuanjie Zheng

**Affiliations:** ^1^Department of Radiology, University of Maryland School of Medicine, 22 S. Greene Street, Baltimore, MD 21201, USA; ^2^Department of Radiology, Affiliated Hospital of Nanjing University of Traditional Chinese Medicine, 155 Hanzhong Road, Nanjing, Jiangsu 210029, China; ^3^Department of Radiology, University of Pennsylvania, Philadelphia, PA 19104, USA

Medical image analysis is performed in order to facilitate medical research and ultimately provide better healthcare. It is critical to the advancement of imaging-based medical research, for example, using magnetic resonance (MR) imaging to probe brain structural and functional changes related to a disease or cognitive process.

This special issue highlights new methods of signal and image processing, computer vision, machine learning, and statistical analysis and their application in medical image analysis. It is organized into two groups of papers: predictive modeling and image processing.

In the first group, we include a set of papers for imaging-derived biomarker detection and clinical decision support systems. The overall theme is how to detect biomarkers characterizing a disease and to build decision support systems using computer algorithms. B. Tay et al. propose a classification scheme for identifying healthy individuals and patients with spinal cord injuries based on fractional anisotropy values obtained from diffusion tensor imaging data. H. Liu et al. address cirrhosis classification based on multimodality imaging and propose a new method to extract texture features for multilabel classification (normal, early, middle, and advanced stages). G.-P. Liu et al. employ deep learning and multilabel learning to construct the syndrome diagnostic model for chronic gastritis. M. Yang et al. describe a method to automatically detect corticospinal tract damage in chronic stroke patients and demonstrate that the detected biomarker is associated with motor impairment. Y. Liu et al. describe a novel machine learning algorithm which combines tract-based spatial statistics and Bayesian data mining to quantify white matter changes in mild traumatic brain injury.

The second group of papers consists of a wide range of medical image processing algorithms, including segmentation, visualization, and enhancement. For brain tissue segmentation, S. Ji et al. describe a multistage method based on superpixel and fuzzy clustering for brain MRI segmentation. Y. Zhong et al. propose a method for regularization of fMRI data to address the limitations of traditional spatial independent component analysis. J. Gao et al. describe a global search algorithm based method to separate MR images blindly. Y. P. Du et al. develop a method to reduce partial volume effect with voxel-shifted interpolation and this algorithm substantially improves the detection of BOLD signal in fMRI. Z. Jin et al. use a high-pass filter based method to enhance the visibility of the venous vasculature and reduce the artifacts in the venography. A framework for tracking left ventricular endocardium through 2D echocardiography image sequence is proposed by H. Ketout and J. Gu. E. Bengtsson and P. Malm review automated analysis of the cell samples to screen for cervical cancer.

This special issue is selective. Among 27 submissions, 12 were selected. It is our hope that this impressive group of papers will help the medical image analysis community in their efforts to advance imaging-based medical research.

*Rong Chen* *Rong Chen* *Zhongqiu Wang* *Zhongqiu Wang* *Yuanjie Zheng* *Yuanjie Zheng*

